# Commentary: Modification of Host Responses by Mycobacteria

**DOI:** 10.3389/fimmu.2017.00466

**Published:** 2017-04-28

**Authors:** Ashutosh Kumar, Mamta Rani, Nasreen Z. Ehtesham, Seyed E. Hasnain

**Affiliations:** ^1^Molecular Infection and Functional Biology Laboratory, Kusuma School of Biological Sciences, Indian Institute of Technology-Delhi, New Delhi, India; ^2^School of Life Sciences, Jawaharlal Nehru University, New Delhi, India; ^3^Inflammation Biology and Cell Signaling Laboratory, National Institute of Pathology, New Delhi, India; ^4^Jamia Hamdard, Institute of Molecular Medicine, New Delhi, India; ^5^Dr Reddy’s Institute of Life Sciences, University of Hyderabad Campus, Hyderabad, India

**Keywords:** *Mycobacterium tuberculosis*, stress adaptation, virulence factors, host pathways, persisters

*Mycobacterium tuberculosis* (*M.tb*), an obligate slow-growing human pathogen, resides within the macrophage after phagocytosis and develops strategies to escape immune surveillance. It can cause active disease or can persist in a latent stage depending on the host immune responses. The mycobacterial cell wall consists of complex layers of arabinogalactan, peptidoglycan, and unusually long branched mycolic acids that are covalently linked with each other. The cell wall of mycobacteria, containing high proportion of lipids, has 15 times less density of pores in comparison to the outer membrane of Gram-negative bacteria (1). This low density of pores might cause more difficulty in absorption of nutrients and could contribute to slow growth of mycobacteria. The other reasons for slow growth are higher GC content of the promoters, differential orientation of the genes in relation to the direction of replication, low RNA/DNA ratio in growing mycobacteria, and presence of a single ribosomal RNA operon present apart from the oriC. Proteins involved in the formation of the substrate-specific energy-dependent transporters ABC transport systems (ATP-binding cassette) are coded by only 2.5% of the *M.tb* genome that is very less compared to 5% in case of the *Escherichia coli* genome (2).

During infection, *M.tb* targets several host pathways such as induction of glycolytic flux (3), endoplasmic reticulum stress (4, 5), disruption of mitochondrial membrane (6), inhibition of apoptosis (7), induction of necrosis (3), phagosome maturation, suppressing host signaling pathways (8), and regulate autophagy to survive within host cell (9). Inside the granuloma, both the mycobacteria and the macrophages survive under stress conditions because of limitation of nutrients. To persist under such unfavorable conditions, both bacteria and macrophages have to conserve their energy by decreasing metabolic rate to allocate available resources toward the production of dedicated stress management proteins. Stress granules formation is a major adaptive defense mechanism through translation repression for stress survival of host cell infected with mycobacteria (4, 5).

Intracellular mycobacteria are found in different vacuolar compartments in distinct physiological state, gene expression, and survival (10, 11). It has been shown that mycobacterial infection activated phagocytes to secrete different cytokines after triggering several host receptors such as type C lectins such as DC-SIGN (12, 13), NOD/NACHT receptors (14), mannose receptors (15), and toll-like receptor 2 (16). Mincle receptor [macrophage inducible Ca^2+^-dependent (C-type) lectin] is a calcium-dependent lectin that is a receptor for mycobacterial cord factor, trehalose-6,6’-dimycolate (TDM). Mincle expression on neutrophils is required for TDM infiltration that binds to both the sugar portion of the glycolipid and the hydrocarbon tail (17).

We have reported several key proteins of *M.tb* that may be functionally important for pathogenesis and survival. Prominent among these are *M.tb* PE/PPE proteins that have multiple role in terms of providing antigenic variation to the pathogen, acting as a molecular switch toward virulence and altering Th1/Th2 host immune response for survival (18–20), immune quorum sensing (21), etc. Interaction of *M.tb* virulence factor RipA with chaperone MoxR1 was required for transport through TAT secretion system (22). Inhibition of *M.tb* chaperonic proteins such as PpiA and PpiB can derail protein folding machinery in *M.tb* (23) and reticence intracellular bacterial survival through alteration of host cytokine profile (24). PpiB also regulates formation of biofilm and can contribute to drug tolerance. Several *M.tb* proteins, such as DATIN, modulate host cytokine profile by interacting with TLR-2 (25), Rv2626c induce the production of pro-inflammatory cytokines through NF-κB (26). Rv2430c induces strong B-cell response (27), while Rv2608 induces different humoral and T-cell response in various categories of TB patients (28). Inhibitors of these proteins can help boost host immune system within host and provide an unfavorable environment for *M.tb* to survive. *M.tb* ORF Rv1475c encoded aconitase is an iron binding protein that has conserved residues of the iron-responsive class of proteins and binds to iron-responsive elements in case of iron depletion (29). It is one of the several *M.tb* proteins identified in 30-day infected guinea pig lungs indicating its role in host–pathogen interaction (30).

There are several proteins present in mycobacteria which help in its survival inside host by slowing down growth at the level of replication (31), transcription (32), and translation (33, 34) (Figure 1). A recent report (35) has described different mycobacterial strategies against host immune responses such as manipulation of the TLR responses, host cytokine responses, antigen presentation by MHCs, inhibition of phagolysosomal fusion, and resistance to reactive nitrogen intermediates. The role of toxin antitoxins systems in mycobacterial growth regulation in unfavorable conditions and role of Clp proteases in reactivation of latent bacilli have been described in detail. It has been shown that arrest of protein synthesis induces formation of persisters (36) that may have similar metabolic and physiological state as the dormant bacteria (37). The persisters are drug tolerant non-grower bacteria, genetically similar sibling of drug susceptible bacteria but physiologically resistant (persistent) against various bacterial drugs (38). Comparative genomic analyses revealed genes associated with survival, virulence, antibiotic resistance, and biofilm formation (39). Many of these genes can act alone or in combination with other genes and thus inhibitors against such genes can prove vital in targeting the virulence and survival of *M.tb*. Drug re-purposing is an emerging strategy where drugs already in clinical use or approved by US FDA for treatment of mental illness, diabetes, malaria, etc. are being tested against some of the pathogen targets described above. Targeting those host cellular pathways that are also commonly utilized by *M.tb* for its survival is yet another mode of developing new drugs.

**Figure 1:**
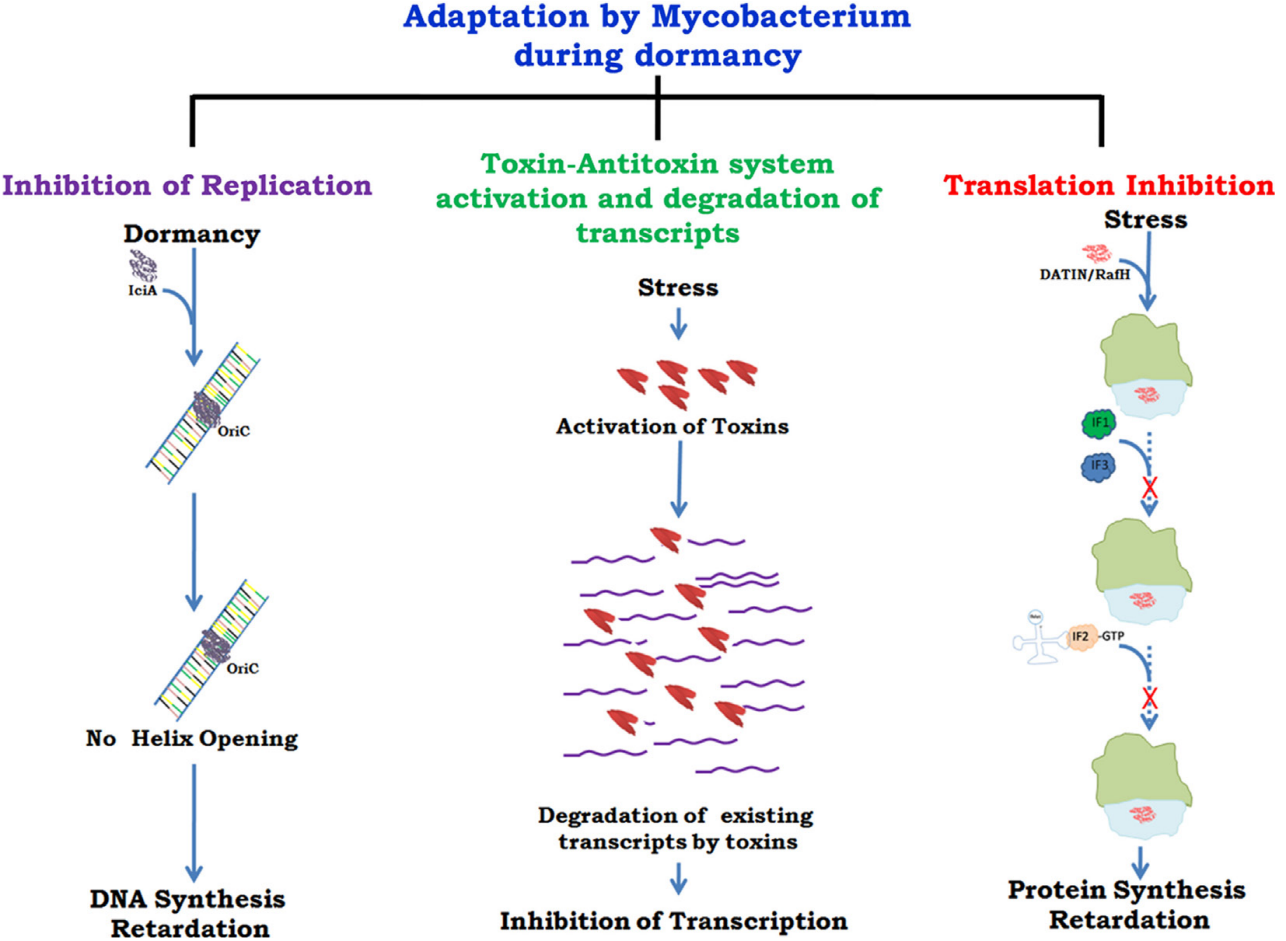
**Growth regulation by *Mycobacterium* for adaptation to stress/dormancy**. *Mycobacterium tuberculosis* (*M.tb*) IciA (inhibitor of chromosome initiation) binds to the A + T rich oriC region of the *M.tb* genome and inhibits helix opening resulting in the arrest of chromosomal DNA replication (31). Activated toxin–antitoxin (TA) systems cleave mRNA to shut down metabolic activity (32). Peddireddy et al. (35) have also described the role of TA systems in *M.tb* and *Mycobacterium smegmatis* to remain in non-replicating phase that help bacteria in antibiotic tolerance. Highly expressed protein DATIN/RafH of *Mycobacterium* inhibits translation by binding with the ribosome under conditions of stress (33, 34). Confirmed and putative roles are indicated with continuous and dashed arrows, respectively.

## Author Contributions

SH and AK conceived the idea behind this commentary; AK and MR wrote the draft; and NE and SH finalized the manuscript.

## Conflict of Interest Statement

The authors declare that the research was conducted in the absence of any commercial or financial relationships that could be construed as a potential conflict of interest.
